# Poly I:C elicits broader and stronger humoral and cellular responses to a *Plasmodium vivax* circumsporozoite protein malaria vaccine than Alhydrogel in mice

**DOI:** 10.3389/fimmu.2024.1331474

**Published:** 2024-04-08

**Authors:** Tiffany B. L. Costa-Gouvea, Katia S. Françoso, Rodolfo F. Marques, Alba Marina Gimenez, Ana C. M. Faria, Leonardo M. Cariste, Mariana R. Dominguez, José Ronnie C. Vasconcelos, Helder I. Nakaya, Eduardo L. V. Silveira, Irene S. Soares

**Affiliations:** ^1^ Department of Clinical and Toxicological Analyses, School of Pharmaceutical Sciences, University of São Paulo, São Paulo, Brazil; ^2^ Laboratório de Vacinas Recombinantes, Departamento de Biociências, Universidade Federal de São Paulo, Santos, Brazil; ^3^ Institut Pasteur São Paulo, São Paulo, Brazil; ^4^ Hospital Israelita Albert Einstein, São Paulo, Brazil

**Keywords:** adjuvant, TLR3-ligand, malaria, antibody-secreting cells, memory B cells

## Abstract

Malaria remains a global health challenge, necessitating the development of effective vaccines. The RTS,S vaccination prevents *Plasmodium falciparum* (Pf) malaria but is ineffective against *Plasmodium vivax* (Pv) disease. Herein, we evaluated the murine immunogenicity of a recombinant PvCSP incorporating prevalent polymorphisms, adjuvanted with Alhydrogel or Poly I:C. Both formulations induced prolonged IgG responses, with IgG1 dominance by the Alhydrogel group and high titers of all IgG isotypes by the Poly I:C counterpart. Poly I:C-adjuvanted vaccination increased splenic plasma cells, terminally-differentiated memory cells (MBCs), and precursors relative to the Alhydrogel-combined immunization. Splenic B-cells from Poly I:C-vaccinated mice revealed an antibody-secreting cell- and MBC-differentiating gene expression profile. Biological processes such as antibody folding and secretion were highlighted by the Poly I:C-adjuvanted vaccination. These findings underscore the potential of Poly I:C to strengthen immune responses against Pv malaria.

## 1 Introduction

Malaria continues to exert a substantial global health burden in tropical and subtropical regions worldwide. According to the World Health Organization (WHO), this disease affected an alarming 3.2 billion individuals in 84 countries in 2021, highlighting that 40% of the world population live in areas at risk of infection. Nearly, 620,000 individuals were killed, especially children, by this illness in the African sub-Saharan region. Among the *Plasmodium* parasites capable of transmitting malaria to humans, five species stand out: *Plasmodium falciparum* (Pf), *Plasmodium vivax* (Pv), *Plasmodium ovale*, *Plasmodium malariae*, and *Plasmodium knowlesi*. Pf, the deadliest of these species, commands attention, but Pv, with its wide distribution and status as the second most prevalent species, presents unique challenges. Contrary to historical perceptions of Pv malaria as benign, recent observations reveal severe symptoms, including cerebral damage, acute kidney injury, anemia, and respiratory complications in afflicted individuals (1). Notably, data from the WHO indicate 4.9 million Pv infections diagnosed annually in Asia, the Western Pacific, the Mediterranean, Central, and South America (2). Adding complexity to the Pv malaria landscape, the parasite can establish dormant hypnozoites in the liver, which may reactivate and lead to recurrent malaria episodes (3).

Malaria elimination and, ultimately, eradication require a multifaceted approach. While vector management and timely diagnostics and treatment remain pivotal, the development of a protective and universally effective malaria vaccine stands as a critical objective long pursued by the scientific community. The circumsporozoite protein (CSP), expressed abundantly on *Plasmodium* sporozoites during the pre-erythrocytic stage of infection, has emerged as a leading vaccine candidate (4). Its central-repeat portion, the most immunogenic region, has demonstrated the ability to generate antibodies capable of neutralizing sporozoites, thereby inhibiting hepatocyte invasion and preventing subsequent morbidity and mortality. Due to the antigen density in the blood-stage of infection and ability to evade infection, residents of malaria-endemic regions tend to develop an increased frequency of antibody-secreting cells (ASCs) and memory B cells (MBCs) specific to non-CSP targets over CSP (reviewed by 5). To overcome this issue, the RTS,S vaccine was conceived. This AS01 adjuvanted-vaccine comprises virus-like particles (VLP), encoded by the hepatitis B virus antigen, expressing different portions of the Pf circumsporozoite protein (CSP): the central-repeat domain and the C-terminal region containing T-cell epitopes. While the full RTS,S vaccination displayed variable efficacy depending on the local parasitic transmission levels, its protection proved to be of limited duration (6). Importantly, high antibody titers specific to the central-repeat region of CSP have been considered RTS,S-derived correlates of protection against Pf malaria (7). Notably, children aged 5-17 months exhibited higher anti-PfCSP IgG titers and protection following a full RTS,S vaccination regimen compared to their 6-12 week-old counterparts (8). Hence, the WHO has approved the implementation of RTS,S vaccination in malaria endemic areas of the African sub-Saharan region (9). However, the central-repeat region of PvCSP has a particularity relative to its Pf counterpart. While Pf sporozoites display a conserved central-repeat region of CSP, polymorphisms have been associated with the Pv sporozoite origin (10–12). Despite this diversity, neutralizing antibody-specific epitopes have been identified within the PvCSP central-repeat region (13, 14), further emphasizing the need for a universal vaccine against Pv malaria.

In the pursuit of a malaria vivax vaccine, two distinct approaches have been explored: PvCSP-derived peptides and virus-like particles (VLPs). The former has demonstrated safety and immunogenicity, stimulating both humoral and cellular responses in a naive population (15). Additionally, the Qβ-peptide platform has induced robust humoral responses and protection against minimal PvCSP peptides (16). On the other hand, VLPs consist of a vector system to display foreign antigens as viral to the host immune system. This strategy has been extensively evaluated, being remarkably effective in generating protection in numerous animal models of infections, including malarial Pv sporozoites. In the latter, immunization with VLP-expressing Rv21 provided a high degree of protection against virulent Pv sporozoite challenges in mice, with Rv21-specific IgG2a antibodies associated with protection, even in the absence of PvCSP-specific T cell responses (17). Moreover, our group revealed that the Poly I:C-adjuvanted immunization with a recombinant PvCSP, encoding its central-repeat region composed by sequences of the 3 major alleles (VK210, VK247, and *P. vivax*-like) and the C-terminal region, elicited high and long-lasting IgG responses against all alleles in mice (18). Overall, this immunization conferred partial protection against parasitic challenges with transgenic *P. berghei* (Pb) sporozoites expressing VK210 or VK247 or *P. vivax*-like PvCSP alleles in their central-repeat region (18–20). Also, the fusion of these 3 PvCSP alleles with the mumps viral nucleocapsid protein formed stable nucleocapsid-like particles (NLP) and protected mice against a malarial challenge with transgenic Pb sporozoites expressing VK210 when combined with Poly I:C (21). However, the precise mechanisms of protection associated with these vaccines remain elusive.

The adjuvant selection is a critical step in vaccine development, with multiple adjuvants described, some advancing to clinical trials, and a few approved for human use. Among them, aluminum salts are widely used adjuvants, comprising amorphous aluminum hydroxyphosphate sulfate, aluminum phosphate, potassium aluminum sulfate, and aluminum hydroxide (including Alhydrogel). Regarding their adjuvant properties, aluminum salts were initially thought to present a slow and continuous antigen release (depot effect) to recruit antigen-presenting cells (22) and eosinophils to the inoculum site (23). Nowadays, it is accepted that their mechanism of action is linked to the activation of NLRP3 inflammasome (24). More specifically, aluminum salts are phagocyted by dendritic cells (DCs) at the injection site, leading to their lysosome blockade and necrosis. Monosodium urate derived from a damage-associated molecular pattern, such as uric acid, can also inhibit DC lysosomes, facilitating the release of antigens and cathepsin B in those necrotic cells. Finally, cathepsin B stimulates the potassium flux that triggers the NLRP3 inflammasome (25–27). Another promising adjuvant is the Poly I:C, a synthetic double-stranded RNA molecule recognized by Toll-like receptor 3 (28, 29) and the cytoplasmic melanoma differentiation-associated protein-5 (MDA-5) (30). This adjuvant stimulates the production of IL-12 and type I IFN, intensifying the innate immunity (31) and vaccine-derived immune responses (32, 33). After interaction, TLR3 dimers cluster along Poly I:C, enabling TRIF recruitment (34, 35) and assembly for the proper downstream signaling through TRAF (36). Furthermore, adjuvants based on the Poly I:C structure have reached clinical trials in humans (37).

In this context, we embark on a comparative analysis, examining the humoral and cellular immune responses elicited by immunizations with yPvCSP-All_CT_ epitopes combined with Poly I:C or Alhydrogel. In addition, we conduct transcriptomic analysis on splenocytes from mice vaccinated with yPvCSP-All_CT_ epitopes or yNLP-PvCSP_CT_ adjuvanted with Poly I:C, or Poly I:C alone, shedding light on the mechanisms underlying these B-cell responses. These findings hold the potential to enhance the development of efficient malaria vivax vaccine formulations and bring us closer to the ultimate goal of malaria eradication.

## 2 Materials and methods

### 2.1 Animals

Six to eight-week-old female C57Bl/6 mice were purchased from the mouse facility at the School of Medicine at the University of São Paulo (USP). The animals were housed under specific pathogen-free conditions at the animal facility of the School of Pharmaceutical Sciences and Biochemistry Institute, USP, with unrestricted access to water and food. All experiments and procedures were performed in accordance with guidelines approved by the local ethics committee (CEUA/FCF 055.2019-P594 and CEUA/FCF 74.2016-P531).

### 2.2 Production of the vaccine antigen

The yPvCSP-All_CT_ epitopes recombinant protein was expressed and purified from *Pichia pastoris* yeast (y) as previously described (18), following good laboratory practices by The Biological Process Development Facility, The College of Engineering at the University of Nebraska (USA).

### 2.3 Immunizations and sampling

To evaluate both humoral and cellular responses, C57Bl/6 mice underwent three intramuscular (i.m.) immunizations with a 2-week interval between each dose. Each vaccine dose consisted of 10 micrograms of yPvCSP-All_CT_ epitopes adjuvanted with 50 micrograms of Poly I:C HMW (Invivogen) or a 1:1 volume of Alhydrogel (Invivogen), totaling 100 microliters. Half of this volume was administered into each thigh muscle. Plasma samples were collected from immunized animals one day before each vaccination dose through submandibular vein puncture. To investigate the Poly I:C effect on the splenic transcriptome of vaccinees, C57Bl/6 mice were immunized three times, two-weeks apart, with 10 micrograms of recombinant protein (yPvCSP-All_CT_ epitopes or yNLP-PvCSP_CT_) adjuvanted with 50 micrograms of Poly I:C HMW (Invivogen) in both cases via the subcutaneous (s.c.) route (38). Spleens were excised after different time points after the 2nd or 3rd vaccine doses for the analysis of cellular responses or transcriptome.

### 2.4 ELISA

Enzyme-linked immunosorbent assays (ELISAs) were conducted to determine titers of plasma IgG antibodies and their isotypes (IgG1, IgG2b, IgG2c, and IgG3) specific to the vaccine antigen (yPvCSP-All_CT_ epitopes). These assays followed a standard operating procedure (SOP) developed by the Clinic Parasitology Laboratory staff (led by Dr. Irene Soares, School of Pharmaceutical Sciences at USP) with modifications. Briefly, ELISA plate wells (Costar high-binding - REF 3590) were coated with 1µg/mL of the recombinant protein used in immunization (yPvCSP-All_CT_ epitopes). Following overnight incubation at 4°C, plate wells were washed four times with PBS and four times with PBS containing 0.5% Tween 20 (0.5% PBS-T20). Subsequently, they were blocked with a 2-hour incubation in blocking solution (PBS supplemented with 10% FBS) at room temperature. Plasma serial dilutions from immunized mice, ranging from 1:100 in blocking solution, were individually added to each plate well and incubated for 90 minutes at room temperature. Plate wells were washed four times with 0.5% PBS-T20, followed by a 90-minute incubation with anti-mouse IgG, IgG1, IgG2b, IgG2c, or IgG3 antibodies conjugated with peroxidase (Southern Technologies, Chattanooga, TN, USA) diluted 1:3,000 in blocking solution at room temperature and in the dark. The final washing steps included four washes with 0.5% PBS-T20 and four washes with PBS. Revelation was carried out using 1mg/mL of O-phenylenediamine (OPD) diluted in phosphate-citrate buffer (pH 5.0) containing 0.03% hydrogen peroxide. The addition of 4N sulfuric acid to each plate well halted the reaction. Plates were immediately read in an ELISA reader (Awareness Technology, model Stat Fax 3200, USA) at an optical density of 492 nm. We considered the end-point titer of a tested sample when its respective dilution presented an optical density (OD) value equal or higher than three-times the blank counterpart.

To estimate the avidity of vaccine-derived antibodies, we conducted an ELISA as described above with the following modifications. After the 90-minute incubation with selected dilutions of day 90-derived plasma samples that generated optical density ratios (450nm/630nm) nearly 1.0, plate wells were washed twice with 0.5% PBS-T20, followed by two washing steps with PBS. Different urea concentrations (6 M, 2 M, and 0.66 M), diluted in PBS, were individually added to each plate well and incubated for 30 minutes at room temperature. Plate wells were washed twice with PBS, followed by the incubation with peroxidase-conjugated anti-mouse IgG antibodies as described above. Values corresponding to the plate wells incubated with no urea represented maximum antibody avidity.

### 2.5 Measuring spleen areas

To estimate the size of the spleen areas, we used the ImageJ software and performed the following steps: 1) A picture of a murine spleen was always taken with a ruler on its side; 2) Image was duplicated, gray-scale transformed (8-bit images), and had its scale adjusted to cm^2^ with the aid of a line of known length; 3) Image was cropped, had its defective region segmented through a manual-adjusting threshold, and the respective remaining area was measured.

### 2.6 ELISPOT

To enumerate antibody-secreting cells specific to the yPvCSP-All_CT_ epitopes recombinant protein used in immunization, the enzyme-linked immunosorbent spot (ELISPOT) assay was employed, following a previously described protocol (39) with modifications. Briefly, 10 µg/mL of the vaccine antigen (yPvCSP-All_CT_ epitopes) were diluted in PBS to coat individual wells of ELISPOT plates (Millipore - cat. MSHAN4B50). After overnight incubation at 4°C, plate wells were washed four times with PBS containing 0.05% Tween 20 (0.05% PBS-T20), followed by four washes with PBS. Plate wells were blocked for 2 hours with RPMI 1640 cell culture medium supplemented with 10% FBS (blocking solution) in a 5% CO_2_ incubator at 37°C. After blocking, the solution was removed, and 10^6^ splenocytes from each immunized mouse were diluted in blocking solution and added to the first-row wells of the ELISPOT plates. Serial cell dilutions, with a 3-fold factor, were performed across the remaining rows, and the plates were incubated overnight at 37°C in a 5% CO_2_ incubator. Subsequently, cells were removed from the ELISPOT plates, and wells were washed four times with 0.05% PBS-T20. An anti-mouse IgG secondary antibody conjugated with biotin (Thermo Fisher Scientific - Cat. B2763), diluted 1:1,000 in PBS containing 0.05% Tween 20 and 2% FBS, was added to the plate wells and incubated for 90 minutes at room temperature. Plate wells were washed four times with 0.05% PBS-T20 and incubated with Avidin-D-HRP (Vector labs), diluted 1:1,000 in 1X PBS containing 0.05% Tween 20 and 2% FBS, for 3 hours in the dark at room temperature. Following incubation, plate wells were washed four times with 0.05% PBS-T20 and four times with PBS. Revelation was carried out by adding the 3-amino-9-ethyl carbazole (AEC) substrate (BD Cat. # 551015) to the plate wells as recommended by the manufacturer. Plate wells were washed with running water and dried before images were obtained using an AID ELISPOT plate reader (KS ELISPOT, Zeiss, Oberkochen, Germany).

### 2.7 Cell staining

After anesthesia and euthanasia, the spleens were removed and macerated in PBS. Red blood cells (RBCs) were eliminated after a 5-minute incubation with the Ack lysis buffer (Lonza) at room temperature. The remaining splenocytes were washed twice with PBS supplemented with 2% FBS (PBS-2% FBS) before staining for distinct B cell subsets. An antibody cocktail was added to the samples for 30 minutes in the dark at 4°C, including anti-B220-APC-Cy7 (clone RA3-6B2 - BD), anti-CD3-FITC (clone 17A2 - Biolegend), anti-F4/80-FITC (clone BM8 - Biolegend), anti-CD138-PE (clone 281.2 - Biolegend), and anti-CD38-APC (clone 90 - eBioscience). Stained cells were washed twice with PBS-2% FBS and fixed with a 4% paraformaldehyde solution. Event acquisition was performed using a FACSCelesta (BD), and data analysis was conducted with FlowJo software.

### 2.8 RNA extraction, cDNA library preparation, and sequencing

Mice immunized with yPvCSP-All_CT_ epitopes or yNLP-PvCSP_CT_ adjuvanted with Poly I:C, or Poly I:C alone had their spleens excised two weeks after the immunization regimen as well as naive mice. Splenic B-cells were purified using MagniSort ™Mouse B cell Enrichment (ThermoFisher Scientific), resuspended in RNAlater solution (ThermoFisher Scientific), and stored at -80°C until use. Total RNA was extracted using the Quick - RNA Miniprep kit (Zymo Research, USA) following the manufacturer’s instructions. RNA integrity was verified for each sample using the Agilent 2100 BioAnalyzer and Agilent RNA 6000 Nano Chips (Agilent). mRNA preparation was performed using the rRNA depletion technique with the Agilent DNA 1000 kit and Agilent 2100 BioAnalyzer equipment. cDNA library preparation and sequencing were conducted by Quick Biology Inc (Pasadena, CA, USA) using the HiSeq 4000 equipment, generating approximately 24 million reads.

### 2.9 Systems biology analysis

Differentially expressed genes (DEGs) were identified using the edgeR program (40). A gene was considered differentially expressed when the p-value was < 0.05 and the fold change (FC) was > 1.5 times compared to naive mice. Functional enrichment analysis utilized the Reactome database (41) through the EnrichR tool (http://amp.pharm.mssm.edu/Enrichr/), with an adjusted p-value < 0.05 indicating statistically significant enrichment. Protein-protein interaction networks were constructed using the NetworkAnalyst 3.0 platform (42) with IMEX interactome curated from the InnateDB database (43), considering only experimental evidence and a 900 confidence-score cutoff. Transcription factor-DEG interaction networks were also defined using the NetworkAnalyst 3.0 platform with the ENCODE ChIP-seq data package following set-up: peak intensity signal <500 and predicted regulatory potential score <1 (through the BETA Minus algorithm). Based on particular parameters, such as degree and betweenness centrality, the resulting networks were visualized with Cytoscape version 3.7.2 (44), and the subnetworks illustrated only immunity-related pathways. DEGs exclusively detected in mice immunized with yPvCSP-All_CT_ epitopes + Poly I:C were highlighted in red, while DEG-associated transcription factors or DEG-relative protein-associated proteins were represented in purple or yellow, respectively.

### 2.10 Statistical analysis

Statistical analyses were performed using GraphPad Prism for Windows, version 6.0 (GraphPad Software, Inc., La Jolla, CA, USA) using a Two-way ANOVA with multiple comparisons through Sidak’s test, computing confidence interval and significance. A p-value (p<0.05) indicated a significant difference between the two groups evaluated.

## 3 Results

### 3.1 Poly I:C-adjuvanted vaccination induced a balanced and durable IgG response compared to Alhydrogel

**Figure 1:**
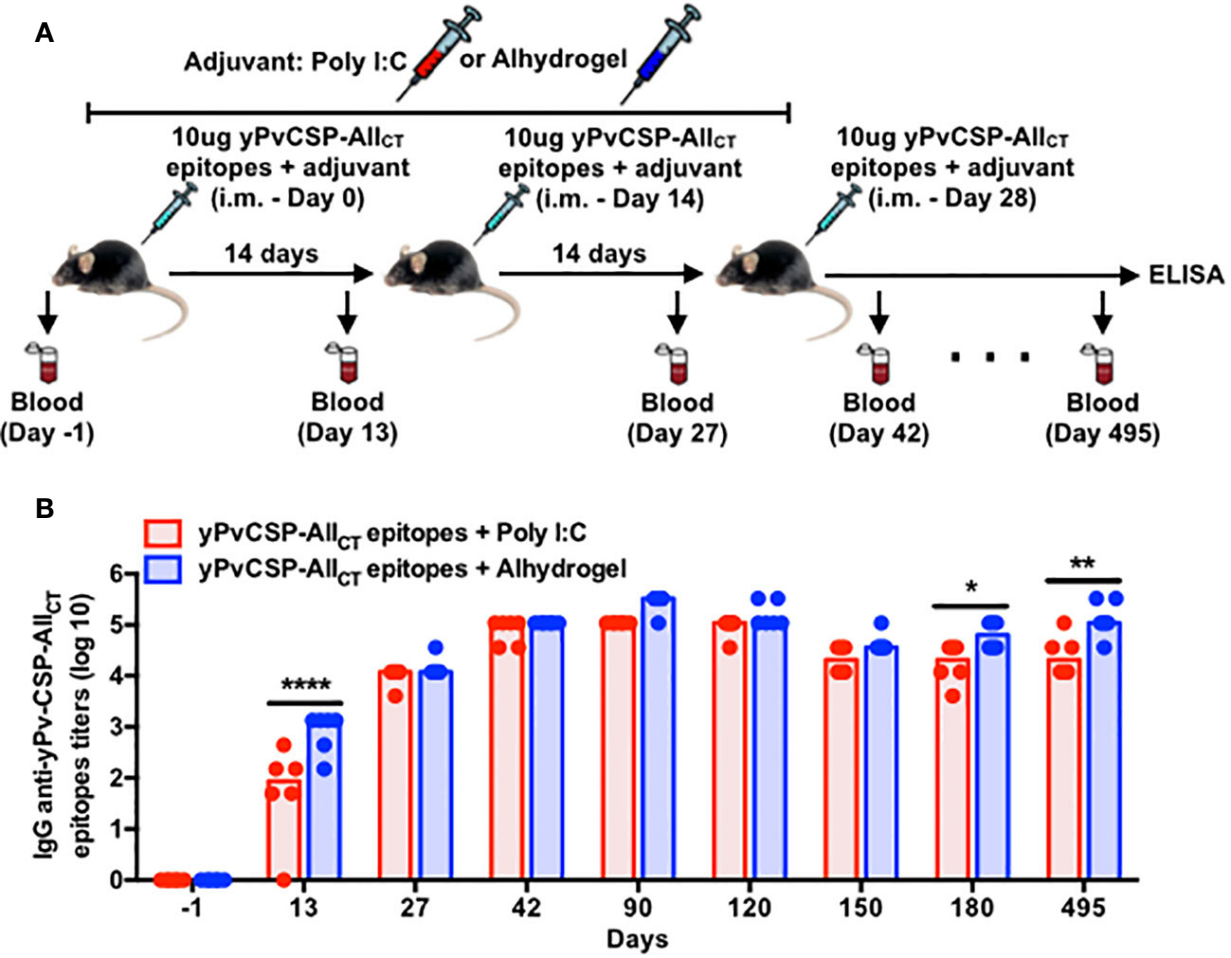
Adjuvanted-malaria vaccine specific to *P. vivax* circumsporozoite protein elicits long-lasting IgG responses in mice. **(A)** Outline of the blood draws and intramuscular vaccination with the recombinant yPvCSP-All_CT_ epitopes protein combined with Poly I:C (n=6) or Alhydrogel (n=6). **(B)** IgG titers specific to the vaccine antigen were measured before, during and after vaccination in plasma samples through ELISA. Dots and columns represent individual values detected for each mouse and median, respectively. Red and blue colors indicate animals immunized with yPvCSP-All_CT_ epitopes + Poly I:C or Alhydrogel, respectively. *p<0.05; **p<0.01; ****p<0.0005.

To assess the immunogenicity of a malaria vaccine targeting PvCSP (yPvCSP-All_CT_ epitopes) with different adjuvants, we conducted a comprehensive study involving 12 C57Bl/6 mice immunized via intramuscular injection with three doses administered at 14-day intervals. The vaccine formulations were combined with either Poly I:C or Alhydrogel as adjuvants. We closely monitored the immune responses of these mice for nearly 500 consecutive days. Plasma samples were collected at various time points before, during, and after vaccination to measure total IgG titers specific to the vaccine antigen using ELISA (**Figure 1A**).

As expected, we observed a significant increase in IgG titers after each vaccination, regardless of the adjuvant used. Interestingly, the presence of Poly I:C as an adjuvant resulted in a slightly faster onset of the vaccine-induced humoral response compared to Alhydrogel. Specifically, Poly I:C-adjuvanted vaccination led to the peak of IgG titers at day 42, maintaining this elevated level until day 120. In contrast, the group that received Alhydrogel had a delayed peak in antibody titers, occurring at day 90 (**Figure 1B**). Importantly, both adjuvants induced IgG antibodies with similar avidity against the vaccine antigen (yPvCSP-All_CT_ epitopes) (**Supplementary Figure 1A**). Antigen-specific IgG titers declined significantly by day 150 but were maintained at a certain level until day 495 for both adjuvants. This suggests that vaccination with either adjuvant can induce durable humoral responses in mice (**Figure 1B**).

### 3.2 IgG isotype profile highlights differential immune responses

**Figure 2:**
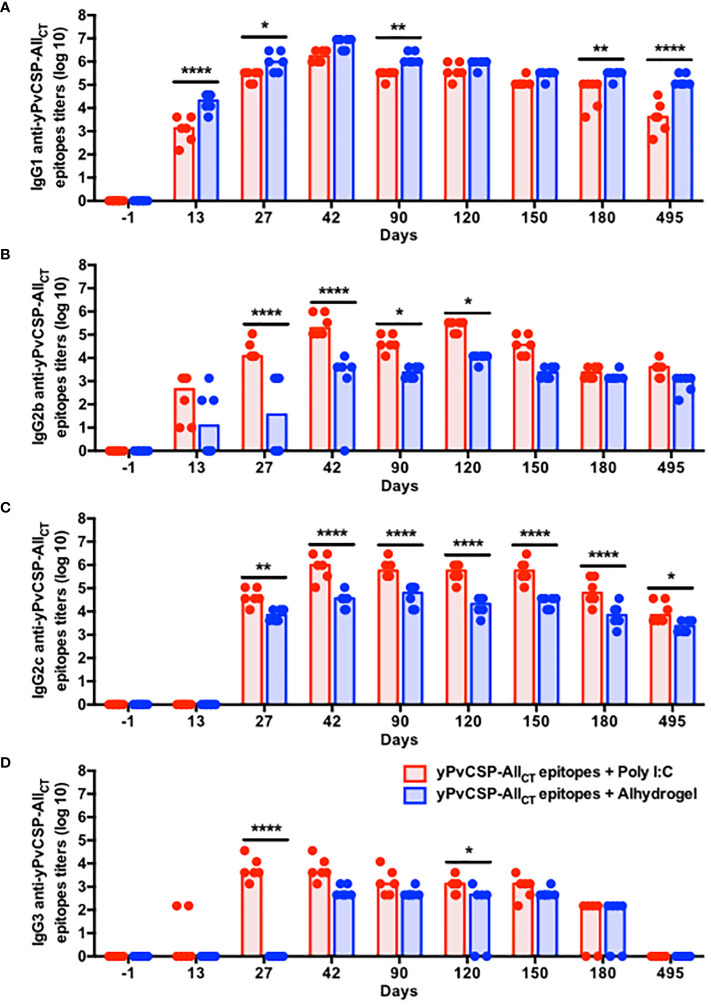
Poly I:C-adjuvanted malaria vaccine triggers a broader isotypic diversification of IgG responses via the intramuscular route in comparison to the Alhydrogel counterpart. Mice were immunized via intramuscular with the recombinant yPvCSP-All_CT_ epitopes protein combined with Poly I:C (n=6) or Alhydrogel (n=6). **(A)** IgG1; **(B)** IgG2b; **(C)** IgG2c; **(D)** IgG3) titers specific to the vaccine antigen were measured before, during and after vaccination in plasma samples through ELISA. Red and blue colors indicate animals immunized with yPvCSP-All_CT_ epitopes + Poly I:C or Alhydrogel, respectively. Dots and columns represent individual values detected for each mouse and median, respectively. *p<0.05; **p<0.01; ****p<0.0005.

The vaccine formulations elicited distinct IgG isotype profiles, shedding light on the nature of the immune response induced by each adjuvant. Notably, IgG1 dominated the humoral response in Alhydrogel-adjuvanted vaccinees, with significantly higher titers observed at day 42 compared to those in the Poly I:C-adjuvanted group (**Figure 2A**). In contrast, while IgG1 displayed the highest titer among the IgG isotypes in Poly I:C-adjuvanted vaccinees, IgG2c, IgG2b, and IgG3 titers followed a hierarchical pattern, peaking also at day 42, with higher magnitudes and a more balanced IgG1/IgG2c ratio (Th1/Th2 profile) compared to Alhydrogel counterparts (**Supplementary Figure 1B**). These antibody titers significantly declined by day 150 (IgG1) or day 180 (IgG2b, IgG2c, and IgG3), becoming undetectable at day 495 (IgG3) in the Poly I:C-adjuvanted group. In contrast, Alhydrogel-adjuvanted vaccinees initiated the decline a bit earlier (day 90) for IgG1, but their remaining IgG isotypes maintained low titers, as observed at day 42, except for IgG3, which was undetected by day 495 (**Figures 2B–D**).

### 3.3 Poly I:C-adjuvanted vaccination enhances the frequency of antibody-secreting cells and memory B cells

**Figure 3:**
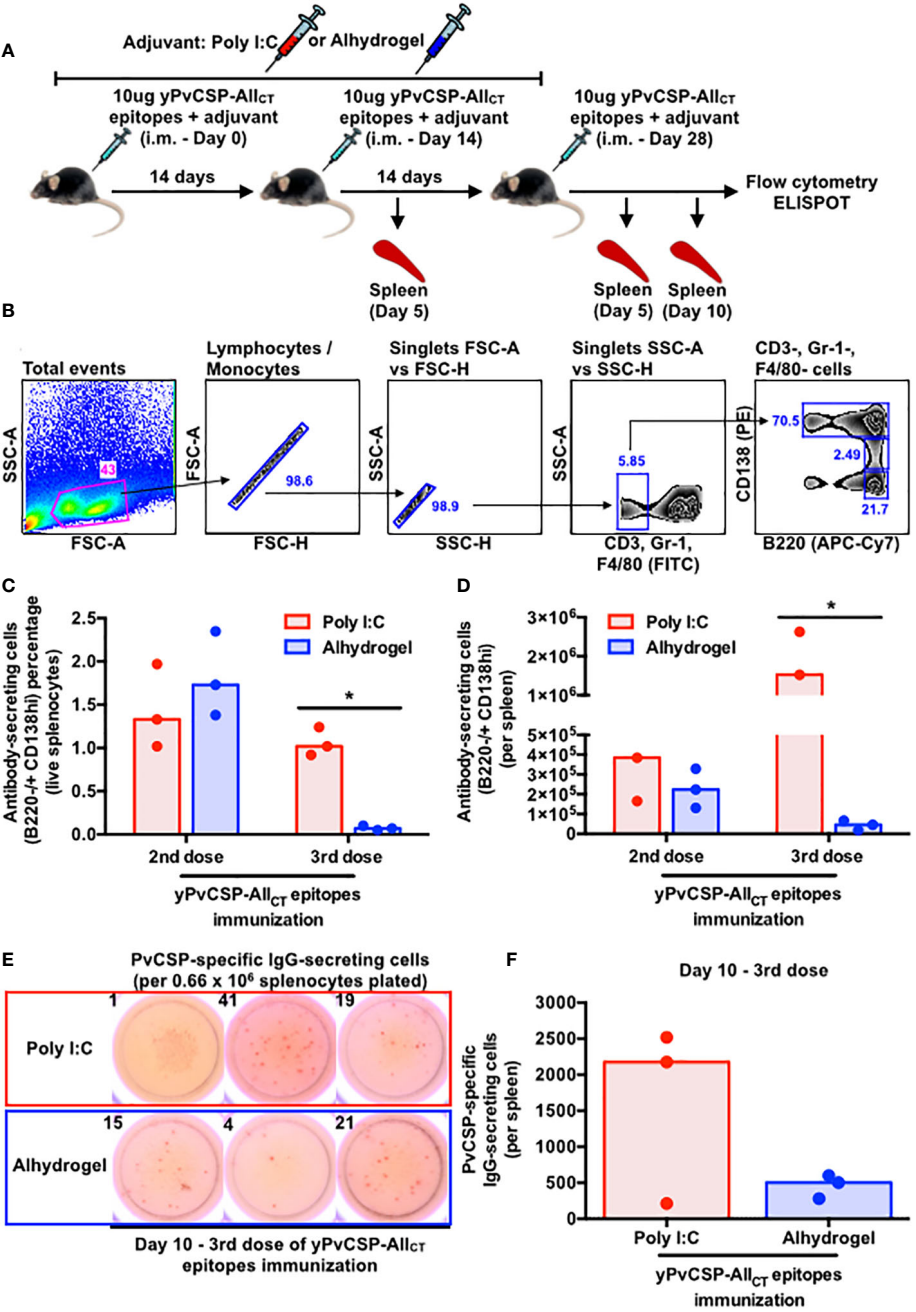
Poly I:C-adjuvanted malaria vaccine induces a more potent antibody-secreting cell response in the mouse spleen via the intramuscular route than the Alhydrogel counterpart. **(A)** Outline of intramuscular vaccination with the recombinant yPvCSP-All_CT_ epitopes protein combined with Poly I:C (red - n=9) or Alhydrogel (blue - n=9) and tissue sampling (n=3 per group per time point). **(B)** Sequential gating strategy to enumerate plasma cells (PCs) through flow cytometry. **(C)** Percentage and **(D)** absolute number of splenic PCs at different time points upon vaccination through flow cytometry. **(E)** Representative images of the ELISPOT results for PvCSP-specific IgG-secreting cells at day 10 of the third vaccine dose (left panel). The numbers on top of each image indicate the quantity of spot-forming cells enumerated per well plated with 0.66 × 106 mouse splenocytes. **(F)** Magnitude of PvCSP-specific IgG-secreting cells per spleen of immunized mice (right panel). Dots and bars represent the totality of splenic PvCSP-specific IgG-secreting cells individually detected for each mouse and median, respectively. *p<0.05

To investigate the impact of adjuvants on the spleen cellularity and on the frequency of B cell subsets, 18 animals were immunized with half receiving each adjuvant (**Figure 3A**). Disregarding the adjuvant used, spleen areas tended to increase from 2nd to final vaccination (day 5). Five days later, those organs returned to initial measures (**Supplementary Figures 2A, B**). Since B cells, involved with antibody responses, are the most abundant immune cells in murine spleens (45), we quantified the frequency and absolute number of several B-cell subsets at various time points following immunization with Poly I:C or Alhydrogel using flow cytometry (**Supplementary Figure 3**). Short-lived plasmablasts (PBs) typically follow specific kinetics upon immunization in different mammals (46–49). In this study, both B cells (B220+) and PBs (B220+ CD138int CD38+) tended to increase in the spleens of mice vaccinated with Poly I:C compared to those receiving Alhydrogel, particularly after boosters (**Supplementary Figures 4A–D**). In contrast, Poly I:C-adjuvanted vaccinees maintained a similar percentage and count of long-lived plasma cells (PCs) at the same period, while Alhydrogel-adjuvanted vaccinees exhibited a significant decrease in both parameters at day 5 after the third immunization (**Figures 3B, C**). To address the specificity of these splenic antibody-secreting cells (ASCs), we enumerated IgG-secreting cells specific to the vaccine antigen at day 10 after the third vaccination using ELISPOT. The Poly I:C-adjuvanted vaccine induced a higher, though not statistically significant, number of IgG-secreting cells specific to the vaccine antigen compared to the Alhydrogel group (**Figures 3D, E**).

The secretion of IgG antibodies relies on the activation and differentiation of follicular B cells, which participate in germinal center (GC) reactions with follicular T cells (TFh) (reviewed by 50), into ASCs. We measured the frequency of important GC players and observed increasing trends in the percentage and absolute numbers of FoBs (B220+ CD23+), GC-Bs (B220+ CD138- CD38- GL7+) and TFh (GC-TFh (CD3+ CD4+ GL7+ CD40L+ CXCR5+) and non-GC TFh (CD3+ CD4+ GL7- CD40L+ CXCR5+)) with the last booster for both adjuvants (**Supplementary Figures 3**, **4E–H**, **5**), although without statistical significance.

**Figure 4:**
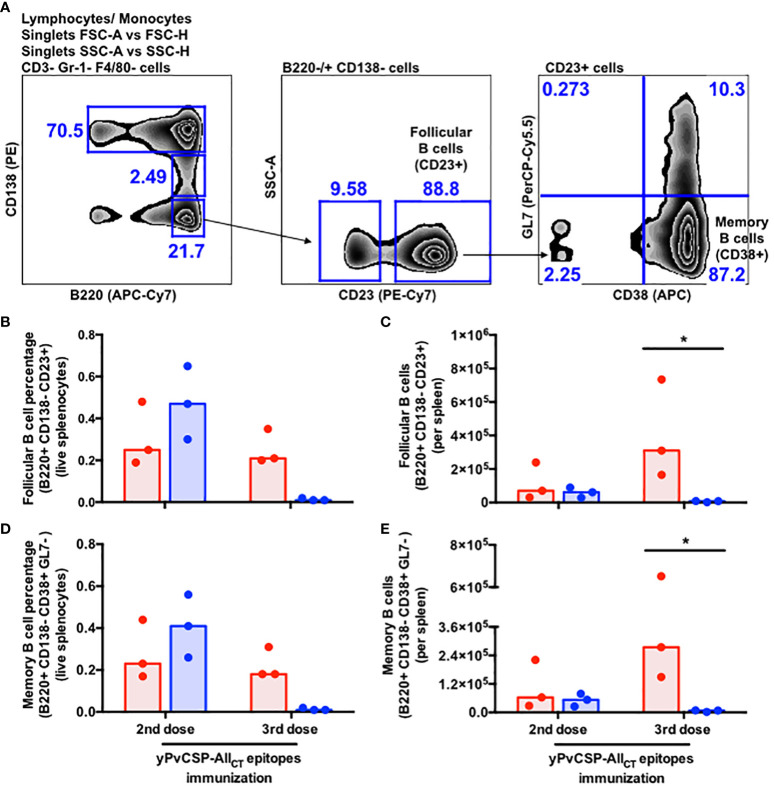
Poly I:C-adjuvanted malaria vaccine induces a stronger memory B-cell response via the intramuscular route relative to the Alhydrogel counterpart. **(A)** Sequential gating strategy to enumerate follicular B cells and memory B cells through flow cytometry. Percentage **(B, D)** absolute number of cells **(C, E)** detected in the spleen of mice at different time points upon vaccination through flow cytometry. Red and blue colors indicate animals immunized with yPvCSP-All_CT_ epitopes + Poly I:C or Alhydrogel, respectively. Dots and columns represent individual values detected for each mouse and median, respectively. * p<0.05

Critical for the durability of vaccine-derived responses and protection, we also evaluated the frequency of memory B cell (MBC) precursors (B220+ CD138- CD38+ GL7+) and terminally-differentiated MBCs (B220+ CD138- CD38+ GL7-) in the spleens of vaccinees. A significantly lower percentage and absolute number of MBC precursors and MBCs were observed in Alhydrogel-adjuvanted vaccinees after the third vaccine dose compared to their Poly I:C-adjuvanted counterparts (**Figures 4A–D**).

### 3.4 Poly I:C-adjuvanted vaccination modulates the expression of genes associated with antibody-secreting cells and memory B cells

**Figure 5:**
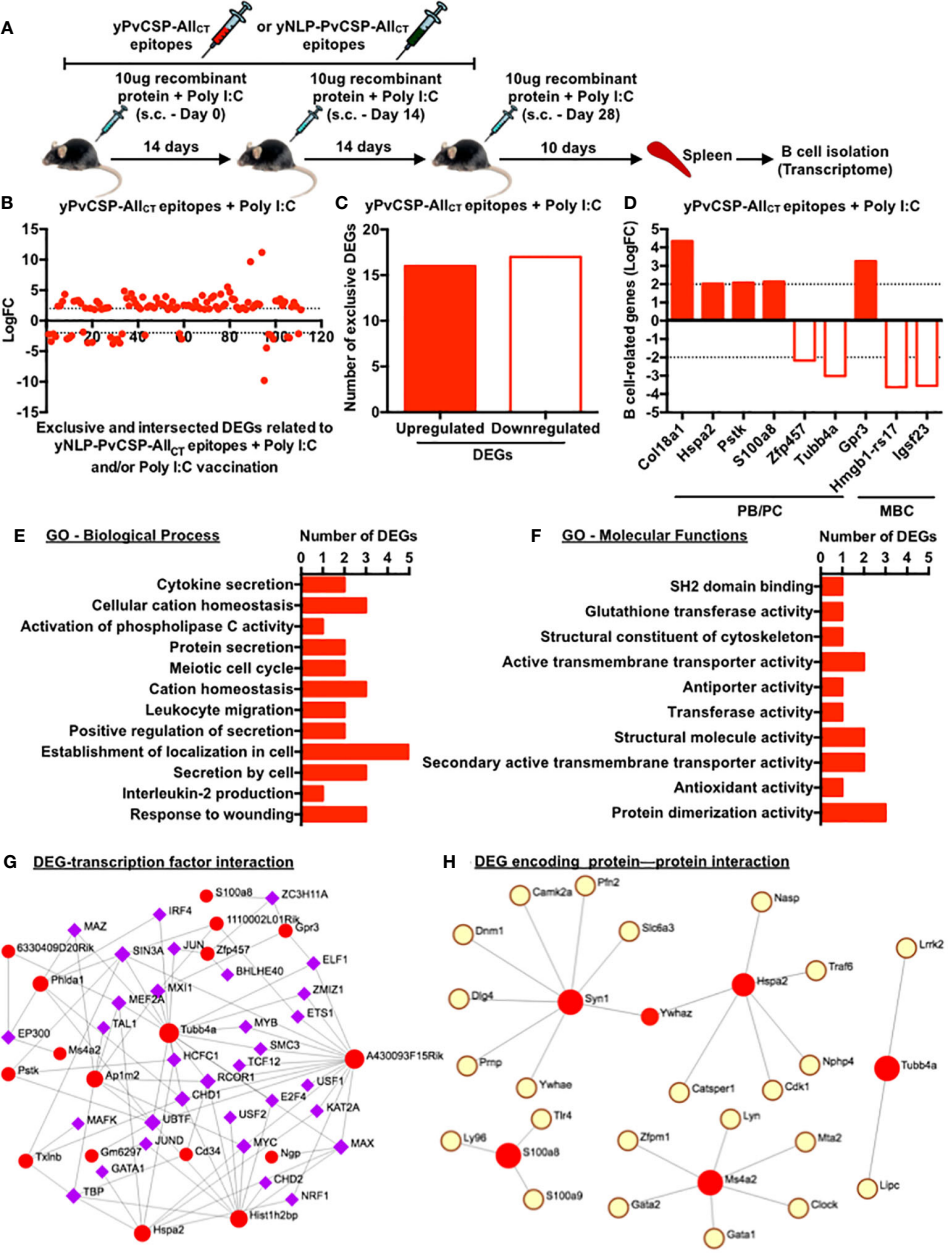
Poly I:C-adjuvanted malaria vaccine elicits modifications in the transcriptome of splenic B cells, enhancing their differentiation into antibody-secreting or/and memory B cells. **(A)** Subcutaneous immunization with yPvCSP-All_CT_ epitopes + Poly I:C, yNLP-PvCSP_CT_ + Poly I:C, or Poly I:C alone, number of doses and their intervals, and euthanasia time for spleen excision, B-cell isolation and freezing for further RNA extraction. **(B)** Log fold-change (FC) of differential expressed genes (DEGs) exclusively induced by the yPvCSP-All_CT_ epitopes + Poly I:C vaccination or mutually induced by yPvCSP-All_CT_ epitopes + Poly I:C and one of the remaining immunizations. **(C)** Number of DEGs exclusively detected in splenic B cells of mice vaccinated with yPvCSP-All_CT_ epitopes + Poly I:C. **(D)** LogFC of DEGs associated with B-cell differentiation into antibody-secreting cells (PB/PC) or memory B cells (MBC) detected upon yPvCSP-All_CT_ epitopes + Poly I:C vaccination. **(E)** Major biological processes and **(F)** molecular functions of splenic B-cell DEGs derived from mice vaccinated with yPvCSP-All_CT_ epitopes + Poly I:C through Gene Ontology analyses. Interaction networks between B-cell-derived DEGs (red dots) elicited upon yPvCSP-All_CT_ epitopes + Poly I:C vaccination with transcription factors **(G)** and DEG-encoding protein with proteins **(H)**. Dotted lines represent LogFC values ≥ -2 and ≤ 2. .

Subcutaneous vaccination with yPvCSP-All_CT_ epitopes combined with Poly I:C demonstrated similar immunogenicity to that obtained via the intramuscular route (data not shown) and protection against transgenic *P. berghei* sporozoites expressing PvCSP alleles (VK210, VK247, or *P. vivax*-like) (18–20). Additionally, a vaccine formulation based on the fusion of the mumps viral nucleocapsid and yPvCSP-All_CT_ epitopes (yNLP-PvCSPCT_CT_) protected mice against parasitic challenges (21). To gain insights into the molecular mechanisms underlying these responses, we compared the splenic B-cell transcriptome of mice vaccinated with yPvCSP-All_CT_ epitopes + Poly I:C, yNLP-PvCSPCT_CT_ + Poly I:C, or Poly I:C alone (**Figure 5A**). This analysis identified nearly 120 differentially expressed genes (DEGs) that were either exclusive to each group or shared among groups (**Figure 5B**; **Supplementary Tables 1**–**3**). Among the 33 exclusive DEGs derived from animals immunized with yPvCSP-All_CT_ epitopes + Poly I:C, 16 were upregulated, and 17 were downregulated (**Figure 5C**). Of these exclusive DEGs, 6 were associated with facilitating B-cell differentiation into ASCs (Col18a1, Hspa2, Pstk, S100a8, Zfp457, and Tubb4a), while others were linked to MBC generation (Gpr3, Hmgb1-rs17, and Igsf23) (**Figure 5D**). Gene ontology analysis indicated that these 33 exclusive DEGs were involved in processes related to cell localization, protein secretion, wound response, and cation homeostasis (**Figure 5E**). At the molecular level, the activities of protein dimerization and transmembrane transport were associated with these DEGs (**Figure 5F**). Gene networks revealed interactions between some of these DEGs, transcription factors (IRF4 and S100a8), or proteins (CamK2a and Cdk1) respectively critical for B-cell differentiation into ASCs or MBCs (**Figures 5G, H**).

## 4 Discussion

The durability of vaccine-induced immune responses is a critical factor in assessing the long-term protective efficacy of vaccination and the potential need for booster doses. Our study demonstrates that both Poly I:C and Alhydrogel adjuvants can elicit robust and long-lasting humoral responses following immunization with the yPvCSP-All_CT_ epitopes formulation. Notably, anti-PvCSP IgG titers persisted for extended periods, declining only after 120 days post-vaccination and remaining stable for almost 350 days thereafter for both adjuvants (**Figures 1**, **2**). This suggests that the number of antibody-secreting cells (ASCs), particularly plasma cells (PCs), generated by yPvCSP-All_CT_ epitopes vaccination with Poly I:C or Alhydrogel does not significantly decrease in the bone marrow of vaccinated individuals within the first year, a phenomenon observed in humans vaccinated against influenza (51). Furthermore, it is plausible that PCs originating from both Poly I:C- and Alhydrogel-adjuvanted vaccinations maintain similar levels of the ZBT720 transcription factor, which is known to sustain humoral responses (52).

Different than the concern raised by the excessive amount of serum anti-CSP antibodies induced by RTS,S vaccination before completing the entire regimen, which hinder the increase of the humoral response (53), all booster doses of yPvCSP-All_CT_ epitopes + Poly I:C or Alhydrogel triggered an enhancement of anti-PvCSP IgG titers (**Figure 1B**). Interestingly, each adjuvant induced a distinct IgG profile specific to PvCSP. The Poly I:C-adjuvanted vaccine triggered a balanced production of PvCSP-specific IgG1 and IgG2c, along with a notable IgG2b response, whereas the Alhydrogel-adjuvanted vaccine was dominated by IgG1 (**Figure 2**). This suggests a potential Th1/Th2 immune profile, which may be advantageous for protection against PvCSP. In comparison to the PfCSP-specific response, the RTS,S vaccination stimulates higher secretion of IgG1, and some IgG3 and IgG2 in humans, being protective when specific to the central-repeat or C-terminal region of the PfCSP. However, these antibody titers significantly wane in less than 8 months and continue to gradually decline in subsequent years. IgG2 and IgG4 have been associated with increased Pf malaria risk and are detected at lower magnitudes than IgG1 and IgG3 (54–56). Regarding the IgG subclasses induced by another malaria vaccine formulation to be implemented (R21 + Matrix-M), they remain elusive in humans. In mice, this latter vaccine elicited higher humoral and cellular responses, culminating with higher protection against transgenic sporozoites compared to R21 + Alhydrogel (57) or R21 alone (58). In this case, the non-protective R21 alone triggered an IgG1-dominated profile (Th2 type) (58) as well as our immunization with yPvCSP-All_CT_ epitopes + Alhydrogel. When other adjuvants, such as SQ or LMQ, were combined with R21, they protected Balb/c mice against a malaria challenge. While the humoral response induced by R21 + SQ was dominated by IgG1 (Th2 profile), the R21+LMQ immunization resulted in comparable titers of IgG2a, IgG1, and IgG3 (balanced Th1/Th2 profile) (58). Notably, our immunization with yPvCSP-All_CT_ epitopes + Poly I:C elicited a similar humoral response, Th profile, and ability to protect against a malaria challenge (18, 19) as R21+LMQ does. Considering that human IgG1 and IgG3 and murine IgG2 are cytophilic, fix complement (59) and interact with Fcγ-receptors on phagocytes, adjuvants capable of triggering distinct Th profiles can eventually facilitate protection against Pv malaria. Moreover, these functional properties of anti-PvCSP antibodies have not been explored yet.

Serum anti-CSP antibodies derived from individuals living in malaria-endemic regions or those immunized with different formulations have been shown to possess neutralizing capabilities against Pf sporozoites (reviewed by 5, 60), reduce the hypnozoite burden, and delay the onset of blood-stage Pv infection (61). Recent molecular dynamics simulations and crystallography analyses suggest that anti-PvCSP neutralizing antibodies efficiently interact with their epitopes, despite the structural disorder of the central-repeat portion of PvCSP (62). However, a non-neutralizing anti-PfCSP monoclonal antibody, isolated from immunized mice, was recently demonstrated to abrogate protection against Pf sporozoites, even in the presence of neutralizing counterparts (63). Given that previous subcutaneous immunizations with yPvCSP-All_CT_ epitopes combined with Poly I:C provided only partial protection in mice exposed to transgenic PvCSP-expressing sporozoites (18–21), it remains unclear whether the vaccine-induced humoral response specific to yPvCSP-All_CT_ epitopes includes non-neutralizing anti-PvCSP antibodies.

Another crucial mechanism of malaria immunity is the opsonization of sporozoites mediated by anti-CSP antibodies. Human anti-PfCSP IgG1 and IgG3 have been shown to interact with neutrophils via FcRIIa and FcRIII, as well as to a lesser extent with monocytes and NK cells, facilitating parasite clearance (64). Antibody-dependent complement activation and fixation are also vital components of effective immunity. Human IgG1 and IgG3 specific to the N-terminal, central-repeat, and C-terminal regions of PfCSP have been demonstrated to fix complement (65). However, it remains unexplored whether anti-PvCSP antibodies induced by yPvCSP-All_CT_ epitopes immunizations can execute these functions, regardless of the adjuvant employed.

Despite the observed discrepancies in IgG responses with the two tested adjuvants, PvCSP vaccination did not result in differences in splenic sizes (**Supplementary Figure 2**). B cells are the most prevalent immune cells within this organ in mice, and various B cell subsets may have their frequencies altered following infection or vaccination. To elicit protective immunity against malaria, a combination of multiple B cell subsets is required. For instance, immunization with irradiated sporozoites (IrSpz), which have CSP as the immunodominant antigen (4), provides protection to several murine models of disease and humans. In mice, the IrSpz-derived response triggers an increased number of CSP-specific plasmablasts and long-lasting germinal center (GC) B cells. The functionality of that cellular response seems to be dependent on T cells, as CD28 KO mice displayed reduced numbers of GC B cells and plasmablasts, and an ensuing higher susceptibility to wild-type (WT) Spz infection (66). The blood stage of malaria is another parameter known to alter the composition of B cell subsets, increasing susceptibility to infection. Mice infected with WT Spz present reduced anti-CSP antibody titers upon the establishment of the blood stage due to an inhibition of the CSP-specific GC B cell response (67). Straight infection with infected red-blood cells also elicits a detrimental GC B cell response (68). Consequently, plasmablasts show a faster decline and only a reduced number of memory B cells (MBCs) are maintained. If mice are treated with atovaquone during the blood-stage of the infection, parasitemia is cleared and animals present a subsequent enhancement in the number of splenic B cells, GC B cells, plasmablasts and anti-CSP antibody titers as observed with IrSPz-immunized mice (67). Notably, a fine tuning for metabolites between plasmablasts and GC B cells seems to occur for prompting protection against malaria. During the blood stage of infection in mice, plasmablasts rapidly proliferate, diminishing levels of blood L-glutamine. Somehow, this scenario delays the proliferation of GC B cells, resulting in reduced numbers of MBCs and plasma cells, and higher-peak parasitemia. On the other hand, if plasmablast depletion or an L-glutamine treatment is done during the beginning of the blood stage of infection, it triggers an effective proliferation of GC B cells and follicular helper T cells, culminating with increased numbers of MBCs and plasma cells, and lower parasitemia peak (69). In this study, Poly I:C-adjuvanted vaccinees displayed significantly higher absolute numbers of PCs, follicular B cells, and terminally-differentiated MBCs compared to Alhydrogel counterparts (**Figures 3**, **4**). Regarding PCs, both qualitative (flow cytometry) and quantitative assays (ELISPOT) exhibited similar kinetics (**Figure 3**), parallel to what has been observed in vaccinated macaques (47) and humans (49). However, the specificities of follicular B cells and terminally-differentiated MBCs induced by our vaccination require further investigation. Moreover, the study sheds light on the cellular aspects of immunity, indicating that Poly I:C may enhance the generation of higher-affinity memory and long-lasting protection against PvCSP relative to Alhydrogel.

The differences in the gene expression profiles of B cells between the two adjuvant groups provide valuable insights into the mechanisms underlying the observed immune responses. Beyond the DEGs identified as B-cell markers in the EMBL-EBI public data repository (**Figure 5D**; https://www.ebi.ac.uk/), several others were exclusively found in splenic B-cells derived from mice of the Poly I:C group, reflecting the robust B-cell response elicited by this adjuvant when compared to Alhydrogel. The enhanced and sustained humoral responses in Poly I:C-adjuvanted vaccinees may be associated with the downregulation of Syn1, which reduces its interaction with CamK2a (**Figure 5H**). This may hinder the transmission of calcium ions within B cells, impacting the regulation of B-cell activation and differentiation (reviewed by 70, 71). Additionally, the downregulation of Hmgb1-rs17 may contribute to the accumulation of splenic PCs and MBCs (**Figures 3**, **4**) by inhibiting B-cell egress from lymphoid tissues, such as Peyer’s patches (72). The regulation of vaccine-derived responses by regulatory T cells (Tregs) could also be affected, as indicated by the downregulation of Gm10408 and Gm14391 (**Supplementary Table 1**), potentially limiting their frequency or functionality in the spleens of Poly I:C-adjuvanted vaccinees (**Supplementary Table 1**). Other downregulated DEGs in Poly I:C-adjuvanted vaccinees represent long non-coding RNAs (Gm6297, 1110002L01Rik, 5830416I19Rik, 6330409D20Rik, and A430093F15Rik), which are more highly expressed in T cells than in B lymphocytes (https://www.ebi.ac.uk/). About the upregulated DEGs, Lilrb4 has been associated with attenuated PRDM1 expression and antibody production. It is possible that the recognition of Poly I:C by TLR3 or MDA5 may maintain Lilrb4 expression at a dysfunctional level. Additionally, Hspa2, which interacts with Cdk1 (**Figure 5H**), is essential for the transcriptional regulation of PC function (73). The positive expression of Col18a1 suggests signaling toward PB formation, particularly when compared to MBCs and naive B cells. Notably, this DEG also interacts with DENV proteins based on disease severity, a condition that leads to a massive PB expansion (74). Phlda1 is a transcription factor with hierarchical expression in naive B cells, followed by MBCs and PBs, and complexes with the IRF4 transcription factor (**Figure 5G**), a fundamental marker for ASC differentiation. S100a8 is highly expressed on the surface of B cells in patients with systemic lupus erythematosus, with its expression decreasing upon disease treatment (75). However, S100a8 displays lower expression in splenic ASCs than in bone marrow counterparts (76). Therefore, the downregulation of genes associated with B-cell activation, calcium ion transmission, and B-cell egress, as well as the upregulation of genes involved in PC formation and ASC differentiation in the Poly I:C group, contribute to our understanding of the enhanced humoral and cellular responses elicited by this adjuvant.

The administration of several vaccine adjuvants, such as products from aluminum hydroxide, has demonstrated to be safe in humans. However, their immunogenicity is long-away off the levels displayed by other adjuvants. For instance, Poly I:C activates immune responses through TLR3 signaling that result in the IFN-α and MDA-5 production (30). In our model, this adjuvant clearly enhances humoral and cellular responses against PvCSP in such levels that immunized mice are protected from malaria challenges (18, 19). Toxicological studies have also supported our vaccination regimen as a safe immunogen (data not shown). However, analogs of Poly I:C have been preferred in clinical trials, such as Hiltonol (also called Poly I:C/L:C), due to its higher stability against serum nucleases present in the plasma of primates, and higher immunogenicity than Poly I:C (77). Thus, the establishment of a clinical trial in which individuals from *P. vivax*-endemic or non-endemic areas be vaccinated with yPvCSP-All_CT_ epitopes + Hiltonol seems to be a critical and subsequent step. An important question to answer is whether the vaccinees would develop high titers of IgG against all repeat domains contained within the yPvCSP-All_CT_ epitopes as observed in mice (18, 19), characteristics that attribute the universality aspect and protection to our vaccine formulation.

In conclusion, our murine model of PvCSP vaccination presents compelling evidence that Poly I:C surpasses Alhydrogel as an adjuvant, eliciting a more balanced and long-lasting humoral response, as well as a more robust cellular memory and an effective response. This provides a strong rationale for further investigation and optimization of adjuvant formulations in the pursuit of a potent and effective vaccine against *P. vivax* malaria. We believe that the insights gained from this comprehensive and longitudinal study will contribute to the accelerated development of a much-needed protective vaccine, ultimately reducing the burden of *P. vivax* malaria in endemic regions and improving global health outcomes.

## Data availability statement

The transcriptomic data presented in the study are deposited in the GEO repository (https://www.ncbi.nlm.nih.gov/bioproject/), accession numbers BioProject PRJNA1092424 (ID 1092424) and BioProject PRJNA839078 (GSE GSE203218).

## Ethics statement

The animal study was approved by the Animal Ethics Committee (CEUA) from the School of Pharmaceutical Sciences, University of São Paulo (CEUA/FCF 055.2019-P594 and CEUA/FCF 74.2016-P531). The study was conducted in accordance with the local legislation and institutional requirements.

## Author contributions

TC-G: Project administration, Writing – review & editing, Methodology, Investigation, Formal analysis, Data curation. KF: Writing – review & editing, Visualization, Software, Project administration, Methodology, Investigation, Formal analysis, Data curation. RM: Writing – review & editing, Methodology, Investigation. AG: Writing – review & editing, Methodology, Investigation. AF: Writing – review & editing, Methodology. LC: Writing – review & editing, Methodology. MD: Writing – review & editing, Methodology. JV: Writing – review & editing, Supervision, Resources, Methodology, Funding acquisition, Formal analysis. HN: Supervision, Software, Resources, Methodology, Investigation, Funding acquisition, Formal analysis, Data curation, Writing – original draft, Visualization, Writing – review & editing. ES: Writing – review & editing, Writing – original draft, Visualization, Supervision, Resources, Project administration, Methodology, Investigation, Funding acquisition, Formal analysis, Data curation, Conceptualization. IS: Writing – review & editing, Writing – original draft, Visualization, Supervision, Software, Resources, Methodology, Investigation, Funding acquisition, Formal analysis, Data curation.

## Supplementary material

Supplementary Figure 1 Similar IgG avidity against the *P. vivax* circumsporozoite protein and differential T-helper (Th) cytokine response patterns triggered by Poly I:C- or Alhydrogel-adjuvanted vaccination. Red and blue colors indicate animals immunized with yPvCSP-All_CT_ epitopes + Poly I:C or Alhydrogel, respectively. Plasma samples (Day 42 **(A)** and Day 90 **(B)**) from mice vaccinated with Poly I:C- or Alhydrogel-adjuvanted malaria vaccine were evaluated for **(A)** IgG and **(B)** IgG1 and IgG2c binding to the vaccine antigen in the presence of different concentrations of urea through ELISA. **(A)** Dots and error bars represent average ± SEM, respectively. **(B)** Dots and columns represent individual values detected for each mouse and their median, respectively. ** p<0.01.

Supplementary Figure 2 Similar spleen area in mice immunized with a *P. vivax* circumsporozoite protein-specific malaria vaccine adjuvanted with Poly I:C or Alhydrogel. Red and blue colors indicate animals immunized with yPvCSP-All_CT_ epitopes + Poly I:C or Alhydrogel, respectively. **(A)** Representative images of murine spleens collected upon first or second boosters. **(B)** Dots and columns represent individual values detected for each mouse and their median, respectively.

Supplementary Figure 3 Sequential gating strategy to enrich distinct splenic murine B-cell subsets: (1) lymphocytes and monocytes; (2 and 3) singlets; (4) B220+ and B220- cells; (5a) Plasma cells (PCs - B220- CD138hi); (5b) B220+ MZBs (CD23-) and FoBs (CD23+); (5c) B220+ cells (CD138- and CD138int); (6a) B220+ CD138- lymphocytes (GCs (GL7+ CD38-), MBC precursors (GL7+ CD38+), and MBCs (GL7- CD38+); and (6b) Plasmablasts (PBs - CD138int CD38+).

Supplementary Figure 4 Similar increasing trend for B cells **(A)**, plasmablasts **(B)**, follicular B cells **(C)**, and germinal center B cells **(D)** in mice immunized with a *P. vivax* circumsporozoite protein-specific malaria vaccine adjuvanted with Poly I:C or Alhydrogel. Red and blue colors indicate the frequency **(A, C, E, G)** and absolute number **(B, D, F, H)** of cells derived from animals immunized with yPvCSP-All_CT_ epitopes + Poly I:C or Alhydrogel, respectively. Dots and columns represent individual values detected for each mouse and their median, respectively. * p<0.05; ** p<0.01.

Supplementary Figure 5 Similar increasing trend for follicular helper T cells in mice immunized with a *P. vivax* circumsporozoite protein-specific malaria vaccine adjuvanted with Poly I:C or Alhydrogel. **(A)** Sequential gating strategy to enrich distinct splenic murine B-cell subsets: (1) lymphocytes and monocytes; (2 and 3) singlets; (4) T lymphocytes (CD3+ CD4+); 5) Activated CD4+ T cells (CD40L+ GL7- and CD40L+ GL7+); 6a) Non-germinal center follicular helper T cells (CXCR5+ GL7-); and 6B) germinal center follicular helper T cells (CXCR5+ GL7+). Red and blue colors indicate the frequency **(B, D)** and absolute number **(C, E)** of cells derived from animals immunized with yPvCSP-All_CT_ epitopes + Poly I:C or Alhydrogel, respectively. Dots and columns represent individual values detected for each mouse and their median, respectively.

Supplementary Table 1 Exclusive differential expressed genes and their respective log fold-change (FC) values detected in splenic B cells upon distinct immunizations.

Supplementary Table 2 Similar differential expressed genes and their respective log fold-change (FC) values mutually detected in splenic B cells upon distinct immunizations. **(A)** yPvCSP-All_CT_ epitopes + Poly I:C and yNLP-PvCSP_CT_ + Poly I:C. **(B)** yPvCSP-All_CT_ epitopes + Poly I:C and Poly I:C alone. **(C)** yNLP-PvCSP_CT_ + Poly I:C and Poly I:C alone.

Supplementary Table 3 Similar differential expressed genes and their respective log fold-change (FC) values detected in splenic B cells upon distinct immunizations.
