# Supplementary figures and images for: Poly I:C elicits broader and stronger humoral and cellular responses to a Plasmodium vivax circumsporozoite protein malaria vaccine than Alhydrogel in mice
Source: Front Immunol. 2024 Apr 8;15:1331474. doi: 10.3389/fimmu.2024.1331474 (PMC11033515; doi:10.3389/fimmu.2024.1331474)

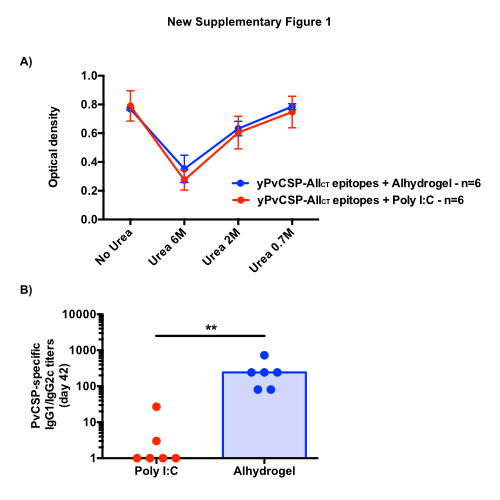

Supplement: Supplementary Figure 1 — Similar IgG avidity against the P. vivax circumsporozoite protein and differential T-helper (Th) cytokine response patterns triggered by Poly I:C- or Alhydrogel-adjuvanted vaccination. Red and blue colors indicate animals immunized with yPvCSP-AllCT epitopes + Poly I:C or Alhydrogel, respectively. Plasma samples (Day 42 (A) and Day 90 (B)) from mice vaccinated with Poly I:C- or Alhydrogel-adjuvanted malaria vaccine were evaluated for (A) IgG and (B) IgG1 and IgG2c binding to the vaccine antigen in the presence of different concentrations of urea through ELISA. (A) Dots and error bars represent average ± SEM, respectively. (B) Dots and columns represent individual values detected for each mouse and their median, respectively. ** p<0.01. [file Image_1.tiff]

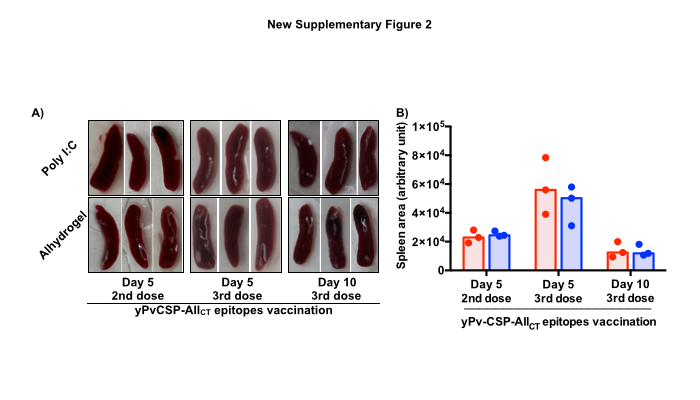

Supplement: Supplementary Figure 2 — Similar spleen area in mice immunized with a P. vivax circumsporozoite protein-specific malaria vaccine adjuvanted with Poly I:C or Alhydrogel. Red and blue colors indicate animals immunized with yPvCSP-AllCT epitopes + Poly I:C or Alhydrogel, respectively. (A) Representative images of murine spleens collected upon first or second boosters. (B) Dots and columns represent individual values detected for each mouse and their median, respectively. [file Image_2.tiff]

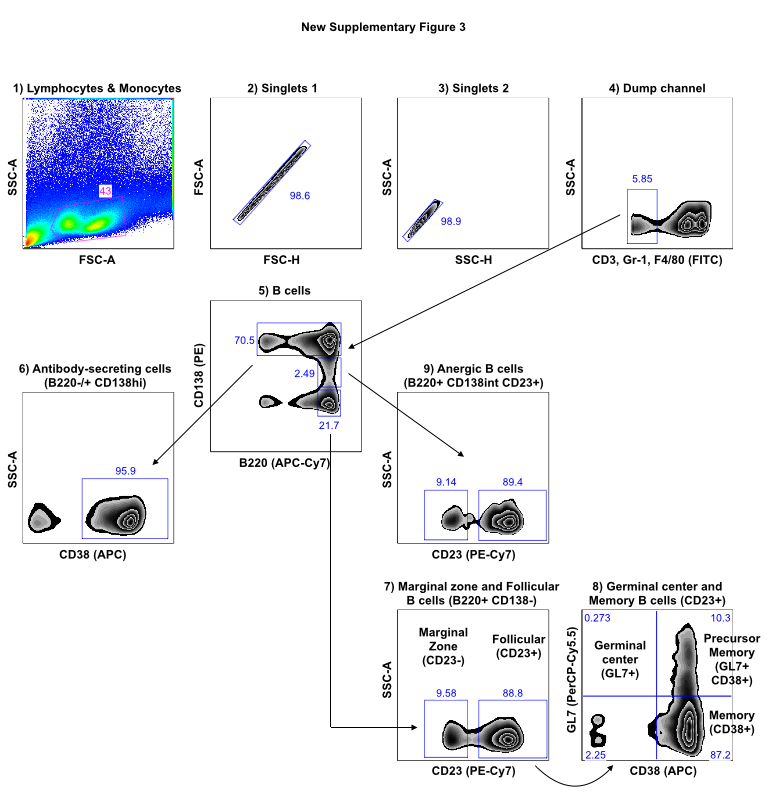

Supplement: Supplementary Figure 3 — Sequential gating strategy to enrich distinct splenic murine B-cell subsets: (1) lymphocytes and monocytes; (2 and 3) singlets; (4) B220+ and B220- cells; (5a) Plasma cells (PCs - B220- CD138hi); (5b) B220+ MZBs (CD23-) and FoBs (CD23+); (5c) B220+ cells (CD138- and CD138int); (6a) B220+ CD138- lymphocytes (GCs (GL7+ CD38-), MBC precursors (GL7+ CD38+), and MBCs (GL7- CD38+); and (6b) Plasmablasts (PBs - CD138int CD38+). [file Image_3.tiff]

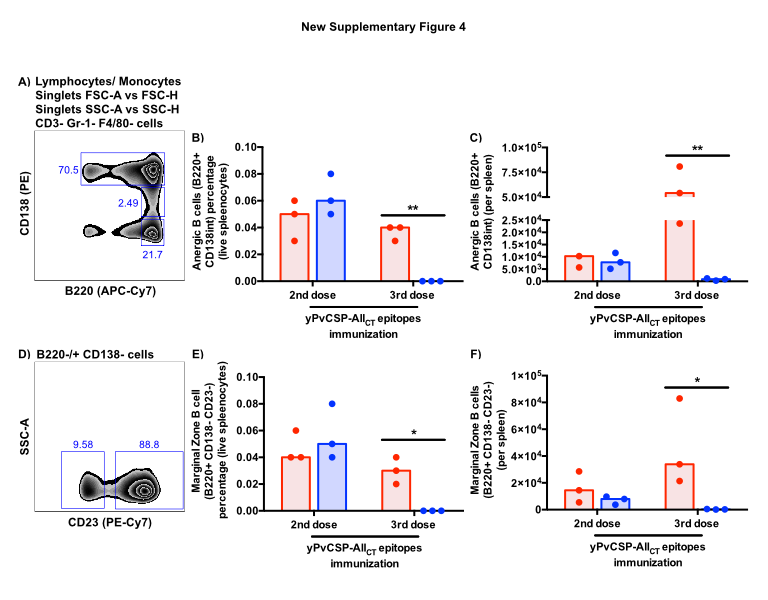

Supplement: Supplementary Figure 4 — Similar increasing trend for B cells (A), plasmablasts (B), follicular B cells (C), and germinal center B cells (D) in mice immunized with a P. vivax circumsporozoite protein-specific malaria vaccine adjuvanted with Poly I:C or Alhydrogel. Red and blue colors indicate the frequency (A, C, E, G) and absolute number (B, D, F, H) of cells derived from animals immunized with yPvCSP-AllCT epitopes + Poly I:C or Alhydrogel, respectively. Dots and columns represent individual values detected for each mouse and their median, respectively. * p<0.05; ** p<0.01. [file Image_4.tiff]

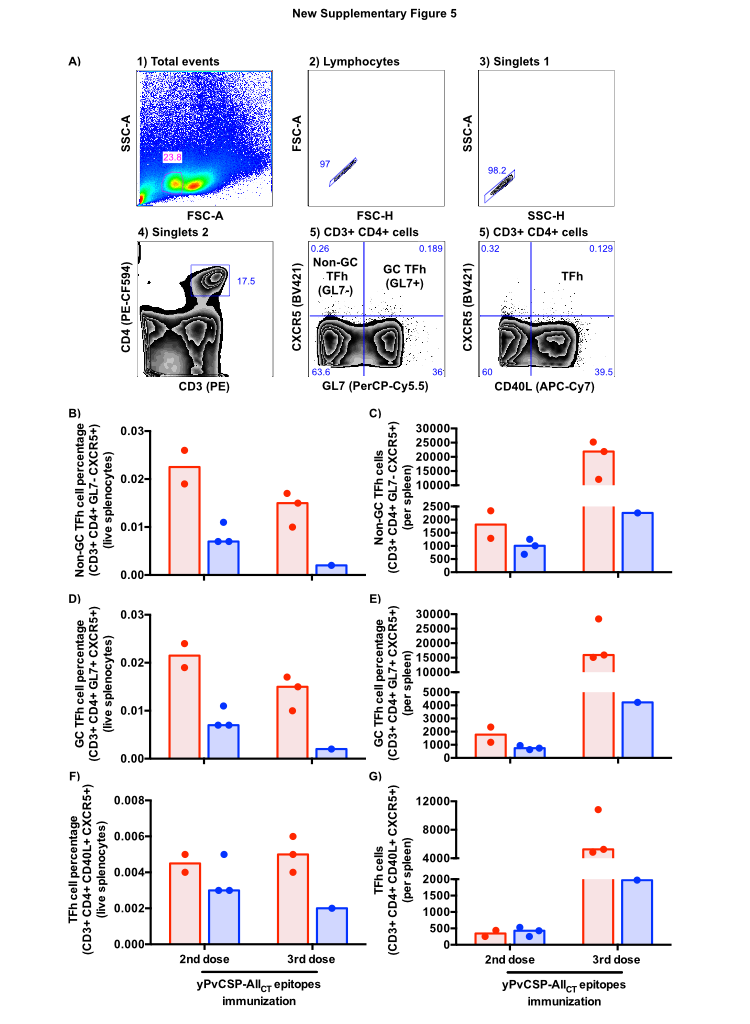

Supplement: Supplementary Figure 5 — Similar increasing trend for follicular helper T cells in mice immunized with a P. vivax circumsporozoite protein-specific malaria vaccine adjuvanted with Poly I:C or Alhydrogel. (A) Sequential gating strategy to enrich distinct splenic murine B-cell subsets: (1) lymphocytes and monocytes; (2 and 3) singlets; (4) T lymphocytes (CD3+ CD4+); 5) Activated CD4+ T cells (CD40L+ GL7- and CD40L+ GL7+); 6a) Non-germinal center follicular helper T cells (CXCR5+ GL7-); and 6B) germinal center follicular helper T cells (CXCR5+ GL7+). Red and blue colors indicate the frequency (B, D) and absolute number (C, E) of cells derived from animals immunized with yPvCSP-AllCT epitopes + Poly I:C or Alhydrogel, respectively. Dots and columns represent individual values detected for each mouse and their median, respectively. [file Image_5.tiff]

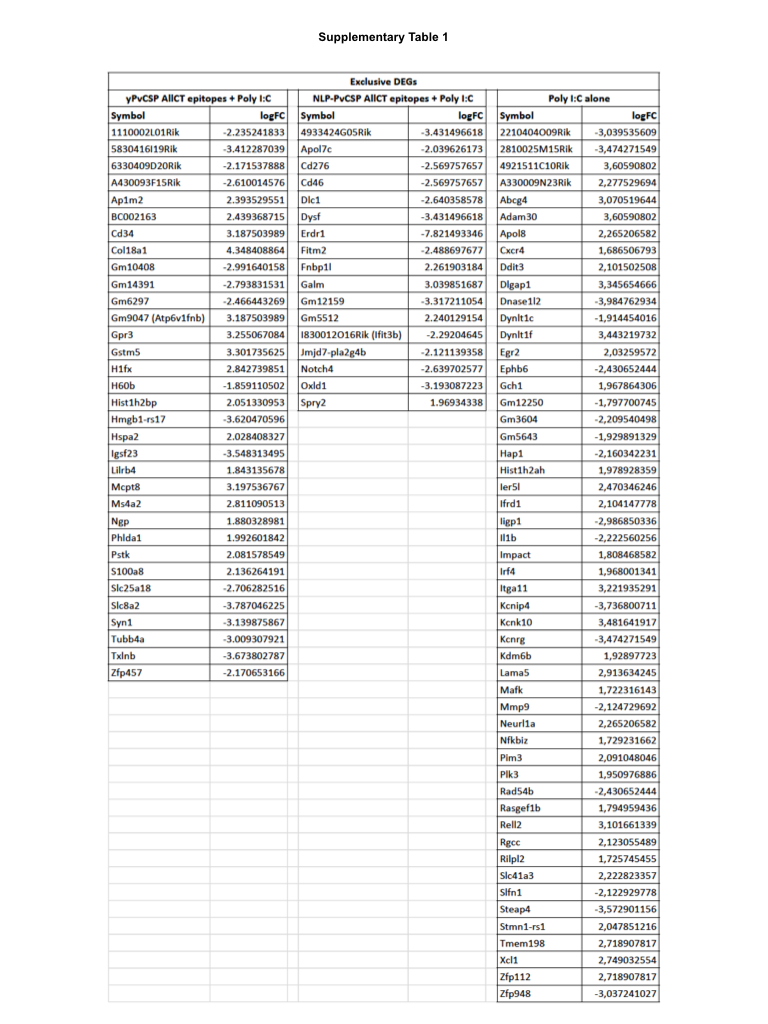

Supplement: Supplementary Table 1 — Exclusive differential expressed genes and their respective log fold-change (FC) values detected in splenic B cells upon distinct immunizations. [file Image_6.tiff]

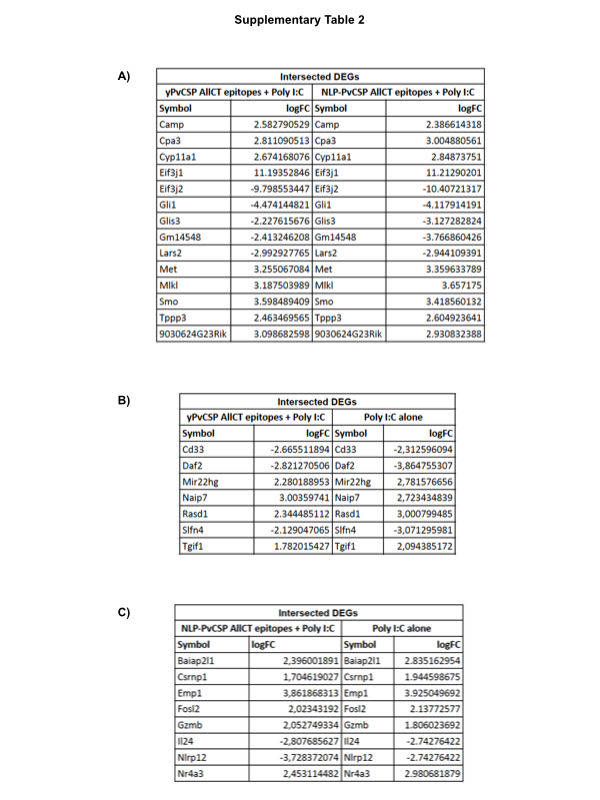

Supplement: Supplementary Table 2 — Similar differential expressed genes and their respective log fold-change (FC) values mutually detected in splenic B cells upon distinct immunizations. (A) yPvCSP-AllCT epitopes + Poly I:C and yNLP-PvCSPCT + Poly I:C. (B) yPvCSP-AllCT epitopes + Poly I:C and Poly I:C alone. (C) yNLP-PvCSPCT + Poly I:C and Poly I:C alone. [file Image_7.tiff]

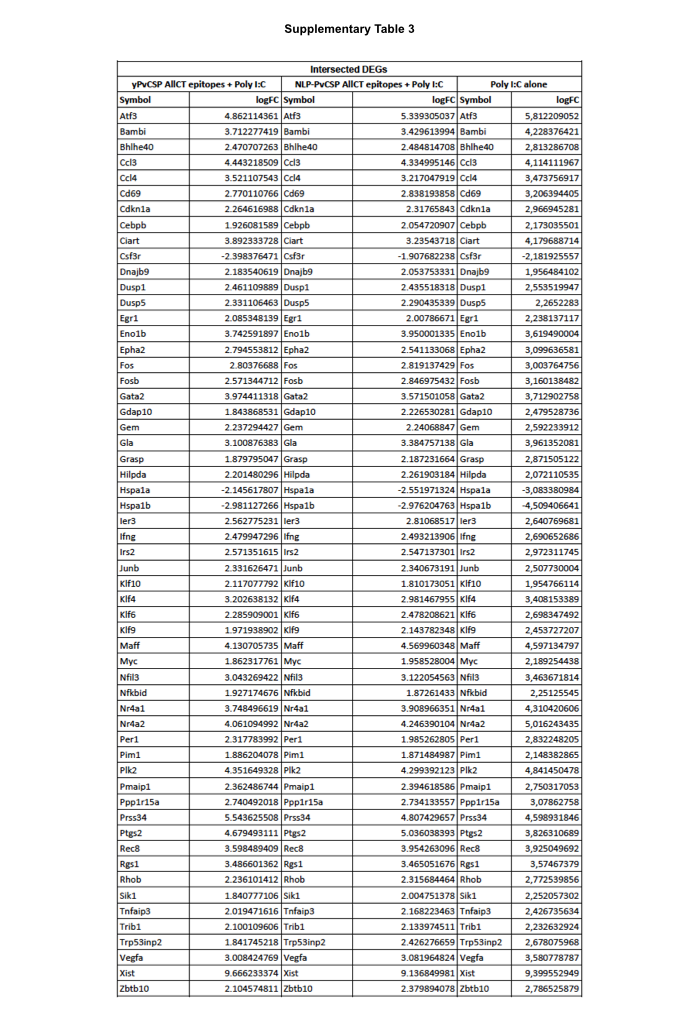

Supplement: Supplementary Table 3 — Similar differential expressed genes and their respective log fold-change (FC) values detected in splenic B cells upon distinct immunizations. [file Image_8.tiff]
